# Basal Lamina Mimetic Nanofibrous Peptide Networks for Skeletal Myogenesis

**DOI:** 10.1038/srep16460

**Published:** 2015-11-10

**Authors:** I. Ceren Yasa, Nuray Gunduz, Murat Kilinc, Mustafa O. Guler, Ayse B. Tekinay

**Affiliations:** 1 Institute of Materials Science and Nanotechnology, National Nanotechnology Research Center (UNAM), Bilkent University, Ankara, Turkey 06800

## Abstract

Extracellular matrix (ECM) is crucial for the coordination and regulation of cell adhesion, recruitment, differentiation and death. Therefore, equilibrium between cell-cell and cell-matrix interactions and matrix-associated signals are important for the normal functioning of cells, as well as for regeneration. In this work, we describe importance of adhesive signals for myoblast cells’ growth and differentiation by generating a novel ECM mimetic peptide nanofiber scaffold system. We show that not only structure but also composition of bioactive signals are important for cell adhesion, growth and differentiation by mimicking the compositional and structural properties of native skeletal muscle basal lamina. We conjugated laminin-derived integrin binding peptide sequence, “IKVAV”, and fibronectin-derived well known adhesive sequence, “RGD”, into peptide nanostructures to provide adhesive and myogenic cues on a nanofibrous morphology. The myogenic and adhesive signals exhibited a synergistic effect on model myoblasts, C2C12 cells. Our results showed that self-assembled peptide nanofibers presenting laminin derived epitopes support adhesion, growth and proliferation of the cells and significantly promote the expression of skeletal muscle-specific marker genes. The functional peptide nanofibers used in this study present a biocompatible and biodegradable microenvironment, which is capable of supporting the growth and differentiation of C2C12 myoblasts into myotubes.

Skeletal muscle is a collection of muscle fibers, which function together as a unit to generate contractile longitudinal forces for physical movement. Muscle cells are the largest cells in the body with long cylindrical shapes and up to a hundred nuclei, due to fusion of many individual progenitor cells. In native skeletal muscle, cells are closely packed and ordered in a parallel orientation^1^. Skeletal muscle tissue constitutes 40% of total body weight and is crucial for physical locomotion. Traumatic injury, tumor excision, congenital defects or myopathies compromise muscle function and mobility and necessitate muscle tissue reconstruction^2^. Muscle stem cell transplantation is a promising treatment for skeletal muscle trauma; however, isolated stem cells drastically lose their ability to form myotubes and function appropriately after *in vitro* expansion. Therefore, antagonism between differentiation and proliferation hampers the therapy^3^. On the other hand, transplantation of non-cultured muscle stem cells to damaged muscle tissue immediately after isolation is quite effective in new myotube formation, although it is required to harvest approximately 3–4 kg of muscle tissue to regenerate 1 × 10^5^ mm^3^ of muscle^4^,^5^. As such, due to lack of donor tissue availability, autologous graft surgery has limited potential. Regenerative medicine is a promising alternative solution for the treatment of myopathies as well as regeneration of age-related muscle wasting in elderly people. It is still challenging to engineer skeletal muscle, yet numerous techniques are being developed^6^,^7^,^8^,^9^,^10^.

Interaction of cells with extracellular matrix plays important roles in regulating cellular behaviors such as adhesion, growth and differentiation. Many of these interactions involve integrin receptors which cluster at specific cell-matrix attachment sites to provide a dynamic connection between extracellular and intracellular environments by organizing the ECM and intracellular cytoskeletal and signaling molecules^11^. Basal lamina is a type of extracellular matrix that surrounds muscle fibers and takes roles in fiber force transmission, repair and maintenance^12^. Myofibers and satellite cells are in close contact with basal lamina through transmembrane receptors, which link them to cytoskeleton and mediate both adhesion and migration during fusion. Fibrous architecture and biochemical components of basal lamina support development and functional integrity of skeletal muscle. Thus, scaffolds mimicking basal lamina are promising candidates for efficient muscle tissue regeneration^13^. ECM proteins such as collagen, fibronectin and laminin or their cell adhesion domains are used to coat surfaces for triggering cell growth and differentiation^14^,^15^,^16^. It was previously shown that laminin has an important role in myogenic differentiation by stimulating proliferation and motility of cells and leading them to bipolar shape of fused cells^12^,^17^. The YIGSR and IKVAV peptide sequences derived from β1 and α1 cell binding domains of laminin, respectively, have been utilized to imitate the natural microenvironment in tissue engineering strategies^18^,^19^,^20^,^21^. The RGD peptide is another important cell adhesion epitope found in many proteins including fibronectin^22^, and was also shown to promote migration and fusion of cells in an *in vitro* myogenesis model system^23^.

Owing to their inherent biocompatibility and tailorable properties, peptide amphiphiles have been demonstrated as versatile tools for designing artificial ECM-like scaffolds^24^. Various shapes including high-aspect ratio nanofibers and nano-networks can be generated through programmed self-assembly driven by multiple types of non-covalent interactions^25^. Peptide amphiphiles enable incorporation of various functional biological signals into their primary sequence and give rise to formation of higher order nanostructures that display complex architecture and biochemical characteristics of native tissue microenvironment under physiological conditions^24^,^26^,^27^. These properties make these molecules ideal candidates to be used as bioactive scaffolds for regenerative medicine and tissue engineering applications.

Here, we show for the first time that bioactive adhesion signals are necessary to regulate cellular processes and induction of myogenesis by using peptide nanofibrous systems. For this purpose, we designed and developed three types of platforms one of which provide only nanofibrous structure with epitope-free peptide nanofibers while others provide both nanofibrous structure and bioactive signals that emulates the natural tissue milieu of skeletal muscle. Peptide sequences derived from the active site of the laminin and fibronectin proteins, which have important roles in muscle regeneration and development, were incorporated into peptide amphiphile nanofibers, so that basal lamina composition of skeletal muscle was mimicked. Combined effect of two adhesive signals were compared with single signal incorporated system as well as epitope free nanofibers, nanofibrous surfaces through analyses of adhesion, proliferation and myogenic potential of C2C12 cells. We also evaluated the differentiation inducing potential of peptide amphiphiles in skeletal muscle regeneration.

## Results

### Adhesion and proliferation of myoblasts on self-assembled peptide amphiphile nanofibers

**Figure 1:**
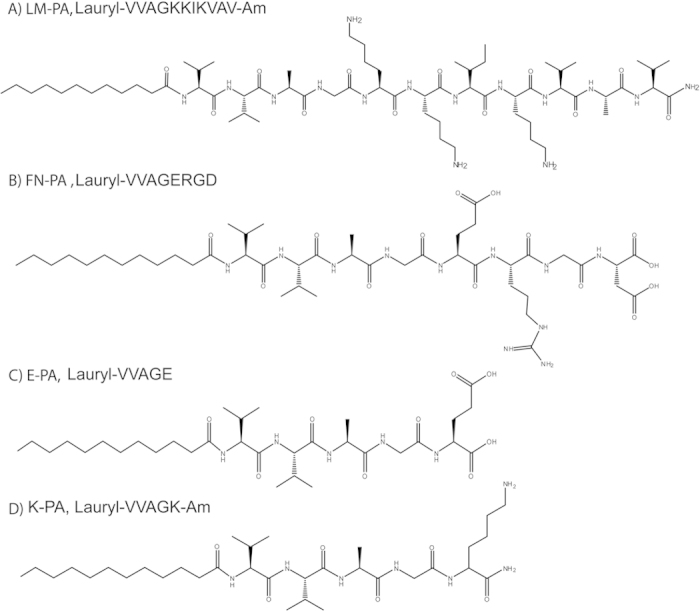
Chemical structures of peptide amphiphile molecules, (**A**) LM-PA, (**B**) FN-PA, (**C**) E-PA, and (**D**) K-PA.

**Figure 2:**
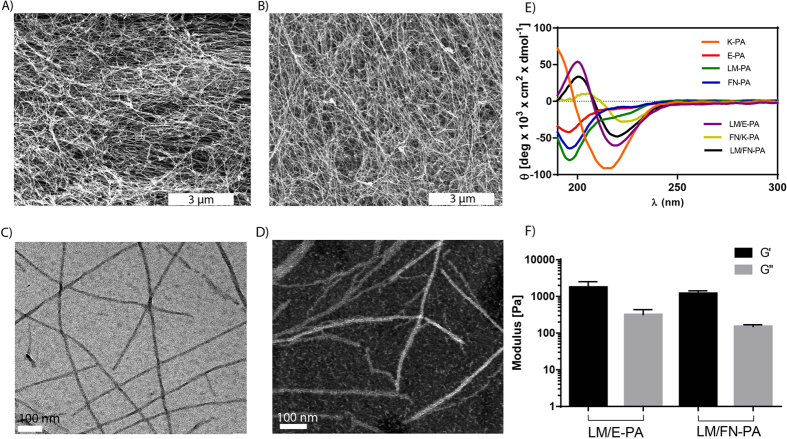
Characterization of peptide amphiphile molecules by using scanning electron microscopy, transmission electron microscopy, rheology and circular dichroism. (**A**) SEM images of LM/FN-PA and (**B**) LM/E-PA gels that reveal the ECM mimicking morphology (Scale bar = 3 μm). (**C**) Representative TEM image of individual nanofibers of LM/E-PA and (**D**) STEM image of LM/FN-PA, (Scale bar = 100 nm), (**E**) secondary structure characterization with circular dichroism, (**F**) storage and loss moduli of mixed PAs.

In this study, laminin-derived sequence “IKVAV” and fibronectin-derived sequence “RGD” were incorporated into peptide amphiphiles (LM-PA and FN-PA, respectively) to generate a myogenic nanofibrous environment suitable for the cultivation of C2C12 myoblast model cells (Fig. 1A,B). LM-PA and FN-PA, which mimic the composition of the basal lamina of muscle cells, were designed to both increase adhesion and survival of progenitor cells and to induce myogenesis. All peptide amphiphiles, including the epitope-free ones, E-PA and K-PA, were synthesized by solid phase peptide synthesis method (Fig. 1C,D). At pH 7.4, LM-PA has a net charge of +3, whereas FN-PA and E-PA bear a −2 charge. Alkyl group induces hydrophobic collapse during self-assembly and the –VVAG peptide sequence is a β-sheet forming unit which is necessary for nanofiber formation. The self-assembly of the peptide amphiphile molecules into nanofibers was triggered by mixing 2 mM of negatively charged and positively charged peptides at pH 7.4, with a volume ratio of 3:2, in order to fully neutralize their charges. Electrostatic interactions between the molecules stabilized the system and resulted in β-sheet formation and nanofiber elongation. Nanofibers were further annealed in order to prevent local supramolecular aggregation of peptide amphiphiles upon mixing and to get a homogenously coated surface. It was previously shown that heating does not significantly affect the β-sheet structure and nanofibrous assembly of the peptide amphiphiles^28^. After annealing, we visualized the nanofibrous morphology and porous structure of the annealed PA networks with scanning electron microscopy (Fig. 2A,B) and individual PA nanofibers with transmission electron microscope (TEM) (Fig. 2C,D). Circular dichroism (CD) analysis was utilized to investigate the secondary structure of the peptide amphiphile molecules. While none of the PAs were found to display an ordered structure by itself, CD spectra of the mixtures of the oppositely charged molecules revealed a minimum at 220 nm and a maximum at 200 nm, which indicate β-sheet secondary structure. The formation of hydrogels by these mixtures (prepared as above) was investigated with oscillatory rheology measurements. LM/FN-PA and LM/E-PA mixtures (Fig. 2F) showed higher storage moduli (G′) than loss (G″) moduli, which was consistent with the formation of a gel. The 1% (wt/v) gels exhibited storage moduli of 1–3 kPa and loss moduli of 100–500 Pa. At the 0.1% strain, both LM/E-PA and LM/FN-PA combinations were similar in terms of mechanical properties. Also oscillatory rheology results of E/K-PA and FN/K-PA are comparable with the other results (Fig. S4A).

**Figure 3:**
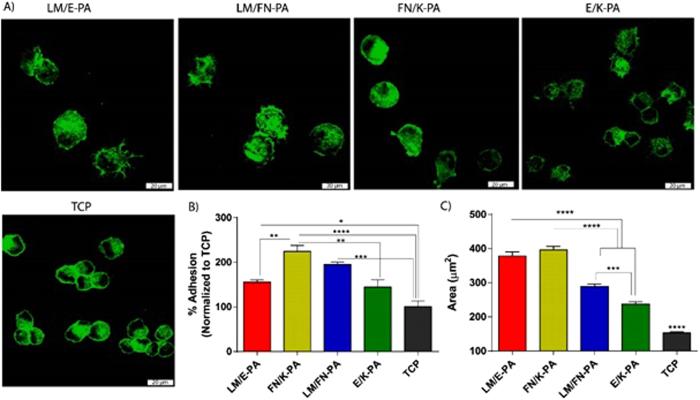
Adhesion and spreading of C2C12 cells on peptide nanofiber networks. (**A**) Confocal images of adhered cells on peptide coated surfaces and tissue culture plate, green represents phalloidin staining and scale bars are 20 μm. (**B**) Relative adhesion of cells after 2 incubation in the presence of BSA and cycloheximide. (**C**) Quantified area from confocal images of adhered cells. Error bars represent mean ± SEM, (*p < 0.05, ****p < 0.0001).

Interactions of cells with their surrounding microenvironment regulate a number of cellular events, including adhesion, proliferation, differentiation and apoptosis. The native extracellular matrix provides the necessary physical and biochemical environment for cells to adhere, grow, differentiate and maintain homeostasis^29^. Therefore, materials resembling native ECM should also support the viability, adhesion and proliferation of the cells. To investigate the effect of peptide nanofiber systems on these cellular behaviors, C2C12 cells were seeded on peptide coated surfaces or uncoated tissue culture plate, and their proliferation and differentiation were evaluated. C2C12 is a mouse myoblast cell line and these cells are commonly used as a model system in skeletal muscle regeneration and differentiation studies. We investigated the initial adhesion behavior of cells to the nanofibrous networks in the presence of BSA and translation inhibitor, cycloheximide, which minimize the interference of endogenous proteins with the adhesion process. After 2 h of culture, cells on nanofibers decorated with bioactive signals, especially on LM/FN-PA and FN/K-PA surfaces, were found to adhere significantly higher than uncoated tissue culture plate (TCP) and non-bioactive nanofiber group. Initial adhesion of the cells and their spreading were better in bioactive signal containing groups analyzed by confocal microscope. Cell area was significantly higher for cells seeded on bioactive peptide nanofibers compared to cells on E/K-PA and TCP. Although, adhered cell number was higher on LM/FN-PA, quantified data showed that cell spreading area was higher on LM/E-PA and FN/K-PA groups (Fig. 3C) after 2 h of adhesion. Confocal images clearly demonstrated larger area of cells cultured on bioactive peptide nanofiber system. Although rigidity is important for myoblast cell cultivation^30^, the initial adhesion of C2C12 cells was not affected by the surface topography or low mechanical strength of nanofibers, as their adhesion was better on nanofibrous network compared to flat TCP surface.

**Figure 4:**
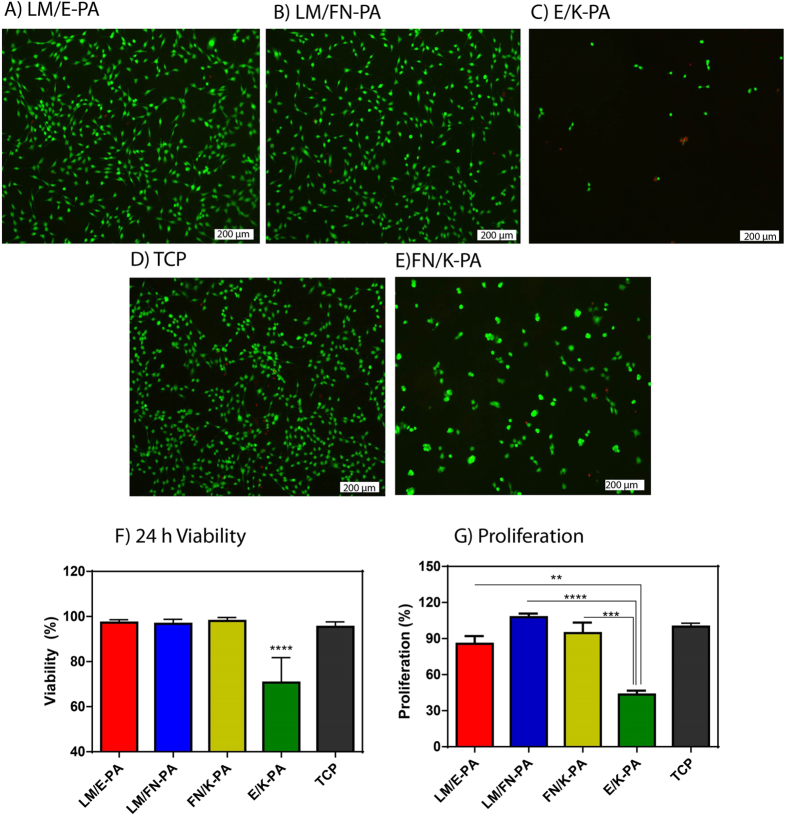
Viability and proliferation of C2C12 cells on peptide nanofiber networks. Live-dead assay of C2C12 cells at 24 h. Dead cells were stained red and live cells were stained green. The scale bar is 200 μm in all images. (**A**) LM/E-PA, (**B**) LM/FN-PA and (**C**) E/K-PA, (**D**) TCP and (**E**) FN/K-PA. (**F**) Relative viability of C2C12 cells on coated surfaces compared to tissue culture plate surface at 24 h. (**G**) Relative proliferation of cells normalized to TCP at 48 h. Error bars represent mean ± SEM, (*p < 0.05, ****p < 0.0001).

24 h viability of C2C12 cells was analyzed with Live/Dead assay, which showed that LM/E-PA and LM/FN-PA nanofibers did not alter viability of C2C12 cells compared to uncoated tissue culture plate surface (Fig. 4A,B,D). Quantitative results of this viability assay demonstrated the biocompatibility of the basal lamina mimetic peptide nanofiber system, which was further confirmed with microscopic observations of the morphologies of C2C12 cells. The spindle-like morphology of C2C12 cells was preserved during their culturing on LM/E-PA and LM/FN-PA nanofibers. However, cells cultured on an epitope-free control peptide amphiphiles (E/K-PA) displayed decreased viability and clustering at 24 h, although they showed initial adhesion to surface (Fig. 4C,F,G, Fig. S3C,D). Overall, cellular viability was supported on bioactive peptide nanofibers and it was comparable with TCP, which indicates the biocompatibility of LM/E-PA and LM/FN-PA peptide nanofibers. Cells on FN/K-PA surfaces showed high viability but displayed clustering similar to cells on E/K-PA. Similar to viability, proliferation of cells was arrested on E/K-PA, as cells cultured on epitope-free peptide nanofibers exhibited decreased BrdU incorporation. Consequently, this combination was not used for further experiments.

When, proliferation profiles were assayed on different surfaces, cells on LM/FN-PA and FN/K-PA coated surfaces proliferated significantly more than cells on LM/E-PA coated surfaces after 48 h (Fig. 4G). This increase in proliferation is likely due to the additional effect of RGD. It is also consistent with the FN/K-PA result which shows highest BrdU incorporation. No significant difference in proliferative capacity was found between the LM/E-PA system and TCP. However, although RGD sequence alone (FN/K-PA) in the absence of IKVAV, showed higher initial adhesion and viability during culture period, cells formed clusters which did not spread on surfaces even at the end of 4 days of incubation (Fig. S5). Cell to cell interaction was probably stronger than cell-matrix interactions and therefore, cells tended to form clusters rather than migrating and adhering onto surface after longer incubation. Hence, this group was also not used in differentiation experiments. As a result of these analyses, we concluded that adhesive and bioactive signals together are necessary for long-term viability and proliferation of cells and hence for inducing differentiation. Fibrous, ECM-mimetic peptide amphiphile system including IKVAV signal either alone or with RGD provides a biocompatible and favorable environment for the healthy growth and proliferation of C2C12 cells.

### Myogenic differentiation of C2C12 cells

**Figure 5:**
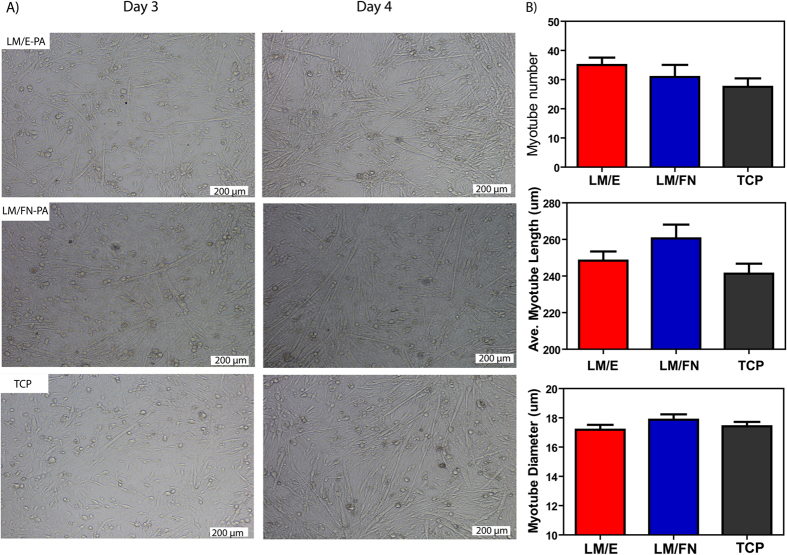
Bright-field images of C2C12 cells cultured on peptide nanofiber networks after 3 days and 4 days of myogenic induction (**A**). (Scale bars = 200 μm) (**B**) The relative myotube number, average length and diameter quantified with Image J. Error bars represent mean ± SEM.

Aggregations of myoblasts mature by fusing and collectively differentiating into multi-nucleated myotubes in a complex process known as myogenesis. During myogenesis, proliferating myoblasts have to exit the cell cycle, contact with each other and align themselves in an end-to-end arrangement in order to fuse with each other^31^. Significant changes in cell morphology are observed during the differentiation process. In this study, induction of differentiation was performed primarily by reducing serum concentrations^8^,^32^. Fusion of the cells was evident within 2 days, and the formation of elongated myotubes was observed after 3 days of incubation with reduced serum medium. Since myotubes have a tendency to delaminate from the surface after long-term incubation^9^, analyses were done at day 3 and 4 to minimize the detachment of cells from the surface. Optical microscopy images clearly showed the formation of myotubes (Fig. 5A, Fig. S6A) after 3 days of incubation. Myotube lengths, diameters and numbers were also evaluated with light microscopy to quantitatively describe myotube morphology and development. Average myotube lengths were around 250 μm, albeit with a wide distribution range between 150–600 μm (Fig. S6). While the cells cultured on LM/E-PA produced a greater number of myotubes compared to cells on TCP, differences regarding myotube numbers, lengths and diameters were not statistically significant between the groups. As such, all groups showed similar profiles in terms of quantitative myotube properties.

The expression of skeletal muscle differentiation markers was investigated by qRT-PCR to further analyze skeletal myogenesis, as well as to understand the effect of peptide amphiphile nanofibers on the expression of vital muscle differentiation genes. Myogenic differentiation involves a sequential and highly ordered series of events, which begins with the expression of myogenic transcription factors and proceeds with cell cycle arrest prior to morphological differentiation^33^.

**Figure 6:**
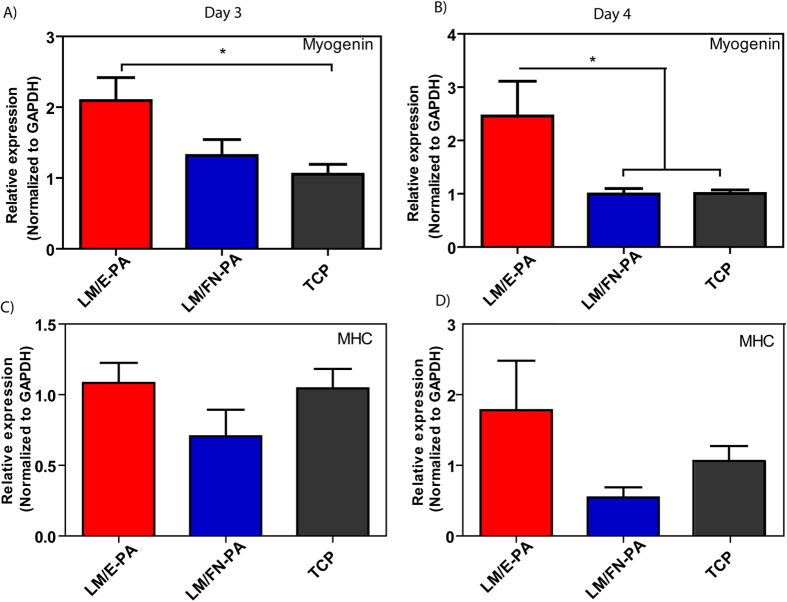
Gene expression analysis of C2C12 cells cultured on nanofiber networks after myogenic induction. (**A**) Myogenin expression at day 3 and day 4. (**B**) Myosin Heavy Chain (MHC) expression at day 3 and day 4. The expression level of each gene was normalized against TCP and GAPDH was used as the internal control. Error bars represent mean ± SEM, (*p < 0.05).

Myogenin is one such myogenic transcription factor, and plays a key role in later stages of myogenesis. Myogenin expression increases in differentiating myocytes, and continues in myotubes. qRT-PCR analysis showed that myogenin expression was increased significantly on LM/E-PA coated surfaces compared to TCP at day 3, although there was no significant difference between the expression levels of LM/E-PA and LM/FN-PA groups (Fig. 6A). However, at day 4, LM/E-PA group had a significantly higher expression of myogenin than both TCP and LM/FN-PA (Fig. 6B). This could be due to the presence of the proliferative RGD sequence on LM/FN-PA. Since myocytes are required to exit the cell cycle prior to differentiation, their proliferation could interfere with the differentiation process. Proliferation analysis results clearly demonstrate that cells cultured on LM/FN-PA have significantly higher proliferation rates than cells on LM/E-PA. Myosin heavy chain (MHC) is another late myogenic differentiation marker, of which expression was analyzed with qRT-PCR. MHC is an important protein for sarcomeric organization of differentiated myotubes. In parallel to myogenin expression, upregulation of MHC expression was also observed in LM/E-PA group compared to other groups, though the difference was not significant (Fig. 6C,D).

**Figure 7:**
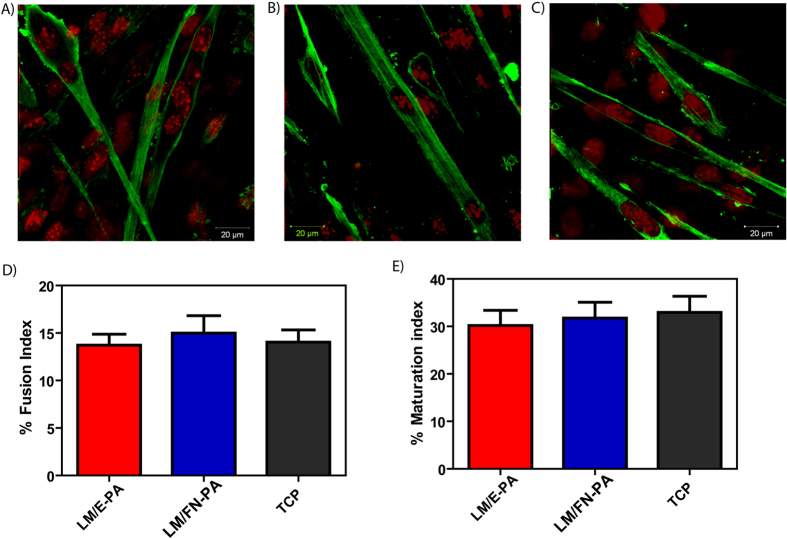
Representative immunofluorescent staining of Myosin Heavy Chain (MHC) and nuclei of myotubes 4 days after myogenic induction on the peptide nanofiber networks (**A**) LM/E-PA; (**B**) LM/FN-PA; and (**C**) TCP (Scale bar = 20 μm). (**D**) The relative fusion index and (**E**) maturation index were calculated from images as reported in materials and methods. Error bars represent mean ± SEM.

Myotubes were stained for analyzing expression of sarcomeric myosin heavy chain protein to characterize and further confirm myogenic differentiation following culturing on bioactive peptide nanofibers at day 4. The differentiation of C2C12 cells towards mature skeletal muscle cells was evaluated by the analysis of stained cells. Confocal microscopy images showing MHC-positive differentiated cells (green) and nuclei (red) are shown in Fig. 7. These results are in good agreement with the previous results, where MHC positive myotubes can be observed in all three groups. Myogenic fusion index, a metric reflecting the number of fused cells per total cell number, was calculated for all groups and found to be *ca.* 15% at day 4, with only slight differences between the groups (Fig. 7D). Maturation index, which denotes the prevalence of myotubes with 5 or more nuclei compared to total myotube numbers, was also measured. 30% of all myotubes were found to be mature in each group (Fig. 7E). Overall, these results demonstrate that IKVAV- and RGD-integrated peptide nanofibers that physically and biochemically mimic the ECM environment are favorable surfaces for growth and differentiation of C2C12 skeletal myoblast cells.

## Discussion

Basal lamina of muscle tissue is primarily composed of laminin, which mediates interactions between cells and extracellular matrix through cell surface receptors. Basal lamina provides a suitable microenvironment for satellite cell residence and plays an important role in regulating regeneration. Therefore, current tissue engineering approaches use ECM proteins or synthetic polymers that are incorporated with peptide sequences derived from ECM proteins to enhance cell adhesion, proliferation and differentiation^34^,^35^,^36^,^37^,^38^. Several previous studies report the use of laminin and fibronectin as adhesion proteins^39^,^40^.

Since fibronectin and laminin are key components of skeletal muscle basal lamina and they have been shown to modulate differentiation of skeletal myoblasts^23^,^41^, in this study, two bioactive peptide sequences, RGD and IKVAV, derived from fibronectin and laminin respectively, were used to fabricate basal lamina-like microenvironment and to enhance myogenesis. By using C2C12 cells as a model system, we generated bioactive peptide nanonetworks from peptide amphiphiles, which favor adhesion, growth and differentiation of myoblasts. LM/E-PA and LM/FN-PA coatings were shown to be biocompatible with C2C12 cells. Incorporation of bioactive signals into the system significantly altered the adhesion and spreading of cells, which were shown by adhesion analysis and confocal imaging. Integration of cell adhesion sequences into the network provided cell proliferation after adhesion and spreading, whereas epitope free control peptide groups showed significantly decreased proliferation. This results show that C2C12 myoblast cells requires cell adhesion sequences for maintaining cellular activities on peptide nanofiber coated surfaces.

While evaluating cell viability and their morphology, our observations showed that cells formed clusters when they were cultured on E/K-PA substrate which indicates stronger cell to cell interaction compared to cell to matrix interaction, which diminishes proper adhesion and further proliferation. In contrast, cell adhesion to matrix was better on bioactive peptide nanofiber group, which resulted in better proliferation and differentiation with no observation of clustering.

Although RGD containing group did not significantly differ from non RGD containing group in terms of cell adhesion after 2 h, this coating enhanced cell proliferation significantly when compared to LM/E-PA coatings, which was in accordance with previously published results that showed enhancing effect of RGD on proliferation of C2C12 cells. However, even though it was previously highlighted that RGD enhances not only proliferation but also fusion and differentiation of C2C12 myoblast through integrin-mediated signaling via focal adhesions^42^, we did not observe a significant difference between myotube formation on LM/FN-PA and LM/E-PA nanofibers.

Myotubes were readily visually apparent under optical microscope in all groups. In addition, RGD containing groups did not show any increase compared to control group in terms of expression of myogenic differentiation markers. In contrast, laminin mimetic-nanofibers (LM/E-PA) significantly enhanced the expression of late myogenic marker, myogenin, in the absence of fibronectin mimetic sequence on day 4. This result indicates that the existence of both sequences at the same time might hamper the myogenic effect of IKVAV sequence. It could also be attributed to increased proliferation signals on LM/FN-PA group due to presence of RGD, which resulted in less cell cycle arrest for differentiation. Although some cells continued to proliferate on LM/FN-PA group, the numbers of cells that were committed to differentiate to myotubes were similar on LM/FN-PA and LM/E-PA groups since there was no statistically significant difference in the number of myotubes between these groups. These results show that laminin derived sequence supports differentiation better when it is presented by itself on the nanofibers, rather than together with fibronectin derived sequence. Overall, our studies revealed that, these bioactive PA nanofiber systems provide the necessary matrix composition to support adhesion, survival, proliferation and fusion of myoblasts towards differentiation in a bioactive epitope selective manner. Especially, laminin derived signal sequence IKVAV, alone, significantly enhances myogenic gene expression. On the other hand, our control peptide nanofiber system supported neither cell viability nor cell proliferation for C2C12 cells. This result further emphasizes the importance of the bioactive signals presented on these artificial scaffolds.

## Conclusion

One of the most important issues in muscle disorders is the infiltration of connective tissue to the site of injury. Presence of connective tissue inside the muscle, which does not possess any contractile capability leaves nonfunctional scar even after healing. Such scars lead to severe weakness in muscles and increase the possibility of fat accumulation within muscle throughout aging. Proper treatment to muscle injuries such as tears or traumas requires use of sophisticated materials to prevent infiltration of connective tissue. To this end, the bioactive peptide nanofiber based system presented in this study constitutes a strong candidacy towards providing myogenic signals to augment muscle regeneration, and thus preventing infiltration of connective tissue. Flexible nature of these systems allows utilization of multiple strategies in a both mechanical and biochemical manner. The present manuscript is the first to demonstrate induction of myogenic differentiation by using laminin mimetic peptide nanofibers. This system was developed to be bioactive and biocompatible by using self-assembled nanofibers functionalized with a myogenic inductive sequence and an adhesion sequence. The presence of laminin-derived signals, in conjunction with the fibrous morphology of the nanofiber network, provides the chemical and structural support necessary for the differentiation of C2C12 cells, while the nanofibrous morphology by itself was not sufficient for neither cell adhesion nor cell viability. Our results show that C2C12 cells demonstrate better adhesion and spreading to bioactive peptide nanofibers compared to epitope-free counterparts and remain viable and proliferative on both of the bioactive peptide nanofiber scaffolds, LM/FN-PA and LM/E-PA. Following induction of differentiation by decreasing serum concentrations, cells differentiated into elongated, multi-nucleated myotubes and showed myogenin and myosin heavy chain expression. With these results it is shown that peptide amphiphile nanonetworks can be used to cultivate muscle myoblast cells and *in vivo* application of gels into damaged muscle could provide resident satellite cell proliferation and differentiation into myotubes. Such properties indicate myogenic potential of these platforms and reveal their potency for muscle regenerative medicine. Due to its biocompatibility, biodegradability and the ease of delivery by injection, our laminin derived signal-incorporating peptide amphiphile system offers a promising platform for future clinical applications.

## Materials and Methods

### Materials

All 9-Fluorenylmethoxycarbonyl (Fmoc) and tert-butoxycarbonyl (Boc) protected amino acids, lauric acid, [4-[α-(20,40-dimethoxyphenyl) Fmoc-aminomethyl] phenoxy] acetamidonorleucyl-MBHA resin (Rink amide MBHA resin) and 2-(1H-Benzotriazol- 1-yl)-1,1,3,3-tetramethyluronium hexafluorophosphate (HBTU) and diisopropylethylamine (DIEA) were purchased from NovaBiochem, Merck and ABCR. All other chemicals for peptide amphiphile synthesis were analytical grade and purchased from Fisher, Merck, Alfa Aesar, or Sigma Aldrich. Other materials used in this study were purchased from Sigma–Aldrich, Invitrogen, Bio-Rad, Fisher, or Merck.

### Synthesis and characterization of peptide amphiphile molecules

Peptide amphiphile molecules were synthesized using solid phase peptide synthesis method with Rink amide MBHA resin and aspartic acid loaded and glutamic acid loaded Wang resin. FN-PA (Lauryl-VVAGERGD) and E-PA (Lauryl-VVAGE) were synthesized on Fmoc-Asp-Wang and Fmoc-Glu-Wang resins, respectively. LM-PA (Lauryl-VVAGKKIKVAV-Am) and K-PA (Lauryl-VVAGK-Am) were synthesized on Rink amide resins. For coupling of amino acids, 1.95 molar equivalents of HBTU and 3 equivalents of DIEA for 1 equivalent of starting resin were used with 2 equivalents of amino acid in 10 mL of dimethylformamide (DMF). Each amino acid coupling took 2 h and removal of Fmoc protecting group was performed with 20% piperidine/dimethylformamide (DMF) solution for 20 min. Lauric acid addition was performed similarly to amino acid coupling except that coupling time was 4 h. In order to acetylate the unreacted amine groups after each coupling step, 10% acetic anhydride–DMF solution was used after each coupling. Dichloromethane (DCM) and DMF were used for washing steps. Peptide cleavage from the resin and deprotection were carried out with 95% cleavage cocktail (95:2.5:2.5 trifluoroacetic acid (TFA):triisopropylsilane (TIS):water) for 2 h at room temperature. Excess TFA was removed by rotary evaporation. Ice-cold diethyl ether was used to precipitate the remaining PA solution overnight at −20 °C. Centrifugation was used to collect the precipitate and ultrapure water was used to dissolve the PA precipitate. Solution was frozen at −80 °C and then lyophilized for 3 days. Lyophilized product was characterized by Agilent 6530 quadrupole time of flight (Q-TOF) mass spectrometry with electrospray ionization (ESI) source equipped with reverse-phase analytical high performance liquid chromatography (HPLC) with Zorbax Extend-C18 2.1 × 50 mm column for basic conditions and Zorbax SB-C8 4.6 × 100 mm column for acidic conditions. In order to purify the peptide amphiphile molecules and remove residual TFA, preparative HPLC system (Agilent 1200 series) was used with a mobile phase of gradient of 0.1% TFA/water and 0.1% TFA/acetonitrile for acidic conditions or 0.1% ammonium hydroxide/water and 0.1% ammonium hydroxide/acetonitrile for basic conditions. 0.1% HCl treatment was used for positively charged peptide amphiphiles. After purification, peptide amphiphiles were lyophilized and stored at −20 °C for further use.

### Nanofiber formation and characterization

Solutions were prepared by dissolving PAs in sterile double distilled water and their pH values were adjusted to 7.4. Each PA molecule was separately heated at 70 °C for 5 min and then mixed at a different volume ratio to have final neutral charge. Following 30 s sonication, mixture of PA combinations were reheated at 70 °C for another 5 min. Plates and coverslips were then coated with PA mixtures. With the annealing procedure, homogenous coating over the surface was achieved. After 30 min of incubation at 37 °C, coatings were left to dry overnight under sterile conditions (Scheme S1).

### Circular Dichroism (CD) analysis

CD (JASCO J815 CD spectrapolarimetry) was used to analyze secondary structures of PA molecules. LM-PA, FN-PA and E-PA were mixed at 2 × 10^−4^ M concentration. pH of the solutions were 7. Peptides were heated, mixed, sonicated and heated again before measurement. Following cooling of mixtures, scanning was done between 190 nm to 300 nm using a digital integration time of 1 s, a band width of 1 nm and with standard sensitivity. Secondary structures of individual PA molecules were also analyzed at 2 × 10^−4^ M concentration. Molar ellipticity was calculated with the data obtained from measurements.

### Mechanical Characterization of PA gels

Oscillatory Rheology (Anton Paar Physica RM301 rheometer) operating with a 25 mm parallel plate configuration at 25 °C was used to show mechanical properties of LM/E-PA and LM/FN-PA gels. Total volumes of 300 μL of 1% (wt/V) PAs were used. After heating, individual PAs were mixed on the center of the plate, whose temperature was set to 70 °C, and incubated for 5 min. Measurement was done after 15 min of cooling, with 0.5 mm gap distance, 100–0.1 rad/s angular frequency and 0.5% shear strain.

### Morphology of Peptide nanofibers

Morphological properties of PA gels were observed with scanning electron microscopy (SEM, FEI Quanta 200 FEG). SEM samples were prepared by mixing 1% (wt/V) LM-PA and FN-PA at 2:3 ratio, and LM-PA and E-PA at 2:3 ratio to have neutral charge. All the samples were prepared with same annealing procedure. Before serial ethanol dehydration step, gels were put onto silicon wafers and incubated for 20 min cooling and stabilization. Then, serial ethanol dehydration step was performed. After gradual ethanol dehydration, gels were dried in a critical point dryer (Tousimis, Autosamdri-815B, Series C critical point dryer) and coated with 5 nm Au/Pd before imaging.

For TEM analysis, sample preparation was done with the same annealing procedure with 1 mM PA concentration. Nanofibers were imaged with transmission electron microscope (TEM) (FEI Tecnai G2 F30 TEM). For sample analysis, samples were prepared with the same annealing procedure. 1 mM E-PA and 1 mM FN-PA were mixed with 1 mM LM-PA in 3:2 volumes and after heating and cooling, mixtures were put on a 200-mesh carbon TEM grid for 1 min followed by 2 wt% uranyl acetate staining for 40 s and drying under flow hood. For E/K-PA 0.5 mM E-PA was mixed with 1 mM K-PA. TEM and STEM images were acquired at 300 kV.

### Cell culture and maintenance

C2C12 cells (ATCC^®^CRL-1772^™^) were cultured in a humidified, 37 °C, 5% CO_2_ incubator using 75 cm^2^ polystyrene cell culture flasks containing High Glucose Dulbecco’s modified Eagle’s medium (DMEM) supplemented with 10% fetal bovine serum (FBS, Gibco), 1% penicillin/streptomycin (P/S) and 2 mM L-glutamine. Passaging of cells was carried out at cell confluency between 50 to 60% to preserve myoblastic characteristics, using trypsin/EDTA. Cells were diluted 1:4 for subculturing. Differentiation induction was carried out after cells became 80% confluent, with high glucose DMEM:Ham’s F10 Nutrient mix supplemented with 2% FBS, 1% penicillin/streptomycin and 10 μg/mL insulin. In all experiments, media were changed every day. Cells were used at passage numbers between 8–10.

### *In vitro* cell culture studies

*In vitro* studies were carried out on LM/E-PA, LM/FN-PA coated well plates and cover slips unless otherwise mentioned. Coating was done as mentioned in the nanofiber formation section. Before coating process, solutions were sterilized under UV for at least 1 h. Gel formation was achieved at 37 °C for 30 min and drying was performed under laminar flow hood overnight. Before experiments, dried plates were further sterilized under UV light for 1 h and washed with medium to remove unbound nanofibers.

Cell viability on the surface of nanofibers was tested with Live-Dead (Invitrogen) viability assay. In this experiment, FN/K-PA control and non-bioactive peptide combination, E/K-PA, was also used. Coated 96 well-plates were used for analysis and C2C12 cells were seeded at a density of 5 × 10^3^ cells/well. After 24 h of standard incubation, plate was centrifuged for 5 min at 2500 rpm to keep dead cells on the surface. Wells were washed with 1× PBS and 2 min centrifugation was done in each step. Calcein AM and Ethidium Homodimer reagents were diluted in PBS so that they had 2 μM and 4 μM concentration, respectively. After subsequent 40 min incubation, cells were observed under fluorescent microscope. Experiment was carried out with n = 4 and, images were taken from 5 different locations per well. Live and dead cells were counted with ImageJ (NIH) software. Viability was assessed by calculating ratio of live cells over total cell number.

Cell adhesion experiment was performed under serum-free conditions. Cells were incubated for 1 h in serum free DMEM medium supplemented with 4 mg/mL Bovine Serum Albumin (BSA) and 50 μg/mL cycloheximide at standard culture conditions before seeding. After 1 h, cells were removed from tissue culture plate by trypsinization and seeded onto the coated 96 well plates. After 2 h incubation in serum-free medium at standard culture conditions, Calcein AM (Invitrogen) staining (2 μM) for 40 min was performed according to the manufacturer’s instructions. Cell adhesion was quantified directly by counting the number of cells using ImageJ program from the images taken with fluorescent microscope. Images were taken from 5 different random locations per well, and experiment was carried out with n = 4. Results were then normalized to tissue culture plate counts.

Cell spreading experiment was also performed under serum-free conditions. Cells were incubated for 1 h in serum free DMEM medium supplemented with 4 mg/mL BSA and 50 μg/mL cycloheximide at standard culture conditions before seeding. After 1 h, cells were removed from tissue culture plate by trypsinization and seeded onto the peptide coated 13 mm glass coverslips at a density of 15 × 10^3^ cells/coverslip. After 2 h incubation in serum-free medium at standard culture conditions, cells were fixed in 4% paraformaldehyde/PBS for 10 min and permeabilized in 0.1% Triton X-100 for 15 min. Samples were incubated with 3% (w/v) bovine serum albumin/PBS for 2 h. After serial washing steps, samples were stained with first 1:250 diluted phalloidine in PBS for 20 min. Coverslips were mounted with Prolong Gold Antifade Reagent (Invitrogen). For cell area and diameter analyses confocal images were taken at 20x magnifications and quantified with ImageJ program.

Proliferation of cells on coatings was assessed using BrdU assay (Roche). Cells were seeded onto PA coated wells and uncoated tissue culture plates (TCP) at a density of 3 × 10^3^ cells/well. Cells were incubated in standard cell culture medium supplemented with 100 μM BrdU labeling solution for 48 h. At the end of the incubation, BrdU incorporation assay was performed according to manufacturer’s instructions. Briefly, cells were fixed with FixDenat for 30 min and anti BrdU-POD solution was added into wells. Following 90 min incubation and tapping, substrate solution was added into wells and proliferation rates of the cells were quantified by measuring the absorbance (370 nm, with 492 nm reference wavelength) with microplate reader.

### Gene expression analysis

Quantitative RT-PCR was used for gene expression analyses. Total RNA was isolated from C2C12 samples using TRIzol (Invitrogen) according to manufacturer’s instructions. Nanodrop 2000 (Thermoscientific) was used to assess the yield and purity of extracted RNA. Primer sequences were designed using Primer3 software (Table S2). The reaction efficiencies for each primer set were evaluated with standard curve using 2-fold serial dilutions of total RNA. cDNA synthesis from RNA and qRT-PCR were performed using SuperScript III Platinum SYBR Green One-Step qRT-PCR Kit (Invitrogen) according to manufacturers’ instructions. Myosin Heavy Chain and Myogenin gene expression profiles were analyzed at day 0, 3 and 4. Reaction conditions were briefly as follows: 55 °C for 5 min, 95 °C for 5 min, 40 cycles of 95 °C for 15 s, 60 °C for 30 s, and 40 °C for 1 min, followed by a melting curve to confirm product specificity. For analysis of the expression data, primary gene expression data was normalized to the expression level of GAPDH. A comparative Ct method was used to analyze the results. Gene expression was normalized to GAPDH and TCP.

### Immunocytochemistry and Confocal Imaging

Antibodies against myosin heavy chain were used for immunocytochemistry as a late marker of myotube differentiation. C2C12 cells were seeded onto PA coated surfaces and glass surface (15 mm) at a density of 8 × 10^3^ cells/cm^2^. After reaching confluency, differentiation was induced with low serum medium. Medium was changed every day. Cells were fixed in 4% paraformaldehyde/PBS for 10 min and permeabilized in 0.1% Triton X-100 for 15 min. To reduce nonspecific binding, samples were incubated with 3% (w/v) bovine serum albumin/PBS for 2 h and treated with 10 μg/mL myosin heavy chain primary antibody (R&D MAB4470) overnight at 4 °C. Then, samples were incubated with Alexa Fluor 488 goat anti-mouse secondary antibody at 1:300 dilution for 1 h at room temperature. Extensive washing with PBS was performed between each step. All samples were counterstained with 1 μM TO-PRO-3 (Invitrogen) and 1:500 diluted phalloidine in PBS for 20 min at room temperature and mounted with Prolong Gold Antifade Reagent (Invitrogen). Negative controls were obtained by omitting primary antibody and incubating with 1% normal goat serum/PBS. Samples were imaged using confocal microscope (Zeiss LSM510).

### Statistical analysis

All quantitative results were expressed as mean ± standard error of the mean (SEM). Statistical analysis was carried out by means of one-way analysis of variance (ANOVA) with Tukey posttest unless otherwise mentioned. A p-value of less than 0.05 was considered statistically significant.

## Additional Information

**How to cite this article**: Ceren Yasa, I. *et al.* Basal Lamina Mimetic Nanofibrous Peptide Networks for Skeletal Myogenesis. *Sci. Rep.* **5**, 16460; doi: 10.1038/srep16460 (2015).

## Supplementary Material

Supplementary Information
