# Supplementary material for: Basal Lamina Mimetic Nanofibrous Peptide Networks for Skeletal Myogenesis
Source: Sci Rep. 2015 Nov 10;5:16460. doi: 10.1038/srep16460 (PMC4639731; doi:10.1038/srep16460)
Supplement: Supplementary Information [file srep16460-s1.doc]

***Supporting Information For***

**Basal Lamina Mimetic Nanofibrous Peptide Networks for Skeletal Myogenesis**

I. Ceren Yasa, Nuray Gunduz, Murat Kilinc, Mustafa O. Guler* and Ayse B. Tekinay*

##
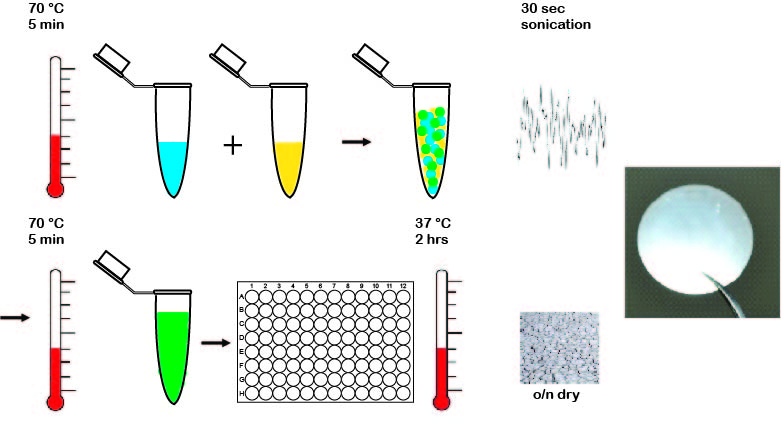


**Scheme S1.** Schematic illustration of nanofiber formation and coating strategy


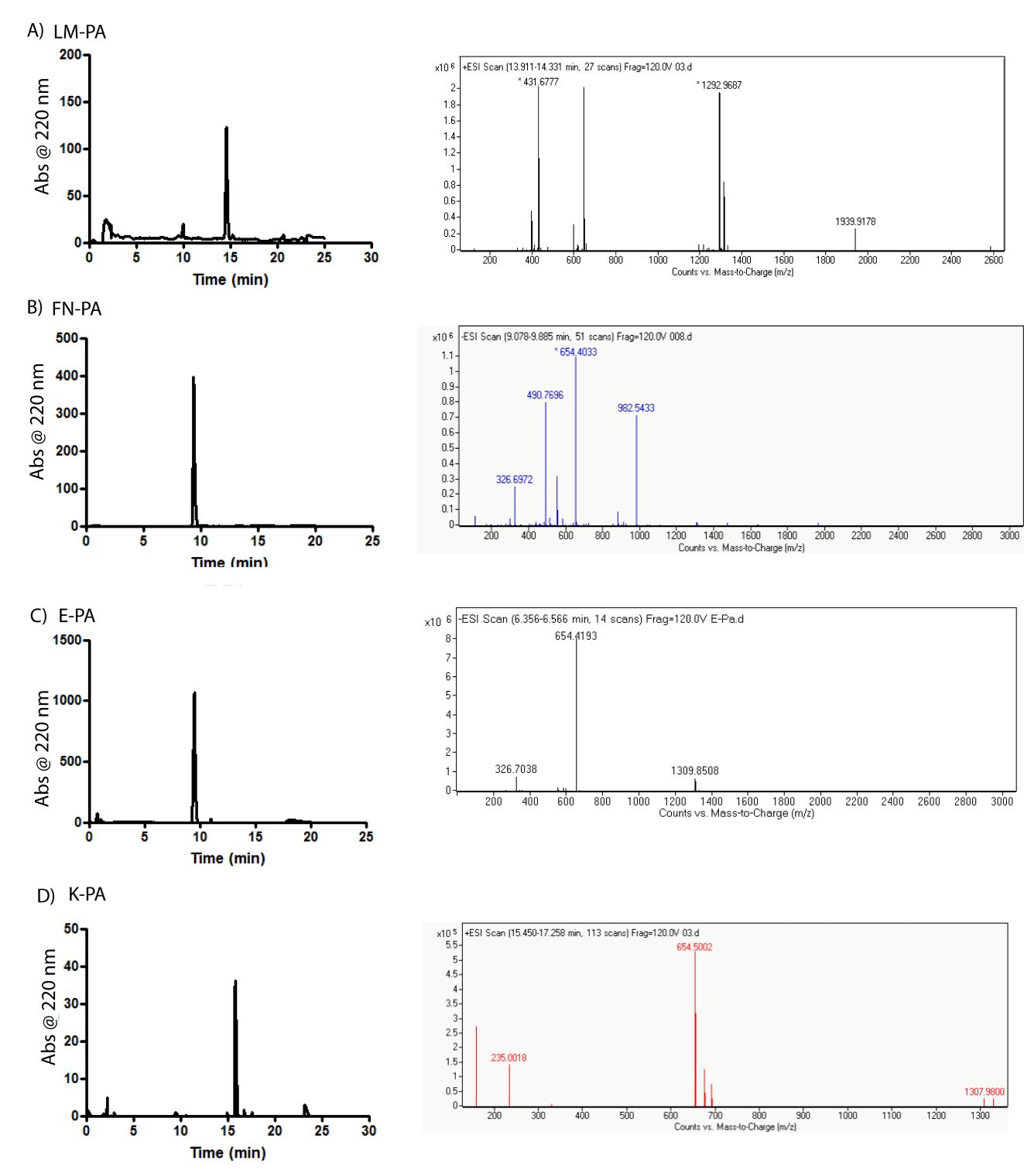


**Figure S1.** Liquid Chromatography and electrospray ionization mass spectra of A) LM-PA, B) FN-PA, C) E-PA and D) K-PA.

**Supplementary Table S1.** Sequences, mass and molecular weight of synthesized peptide amphiphiles

| Name | Sequence | Exact Mass | Molecular Weight |
| --- | --- | --- | --- |
| LM-PA | Lauryl-VVAGKKIKVAV-NH_2_ | 1291.93 | 1292.74 |
| FN-PA | Lauryl-VVAGERGD | 983.57 | 984.15 |
| E-PA | Lauryl-VVAGE | 655.42 | 655.82 |
| K-PA | Lauryl-VVAGK- NH_2_ | 653.48 | 653.90 |

Peptide amphiphile nanofibers are self-assembled through β-sheet formation and hydrophobic interactions upon charge neutralization. In this study, annealing procedure was used to prepare homogenous and stable peptide nanofibers, and this method was compared with normal preparation conditions. Circular dichroism and SEM analysis showed that annealing does not disturb β-sheet structure and nanofibrous assembly. However, resulting hydrogel was mechanically stronger when prepared through heat and cool cycle.

When compared to CD results of unheated samples (Fig. S2B), signal intensity decreased in annealed LM/E-PA mixture but still showed a strong β-sheet conformation. On the other hand, annealed LM/FN-PA showed similar signal intensity to unheated LM/FN-PA. Overall, the CD analyses suggest that the annealing procedure does not alter the β-sheet structure (Fig. 2E).


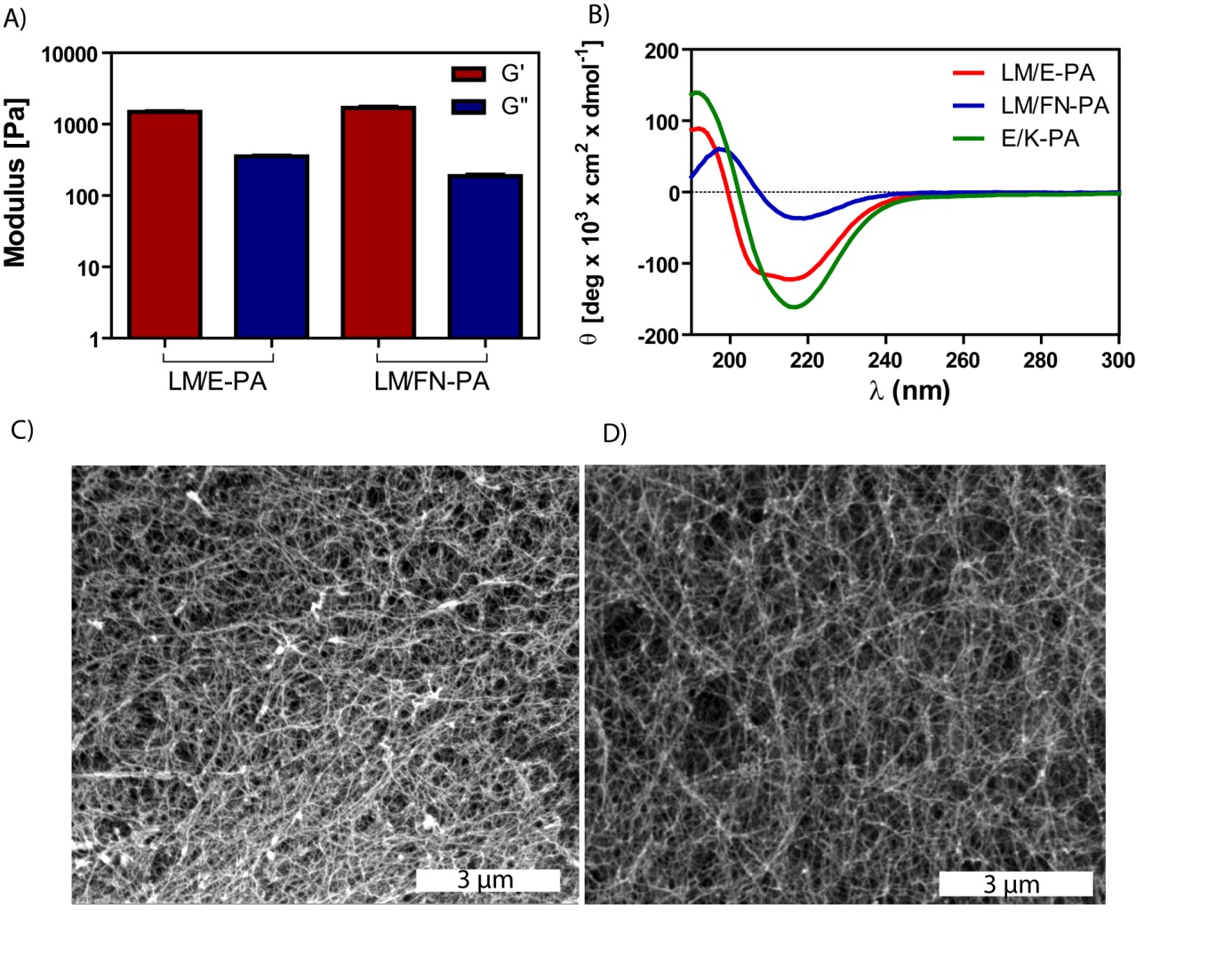


**Figure S2.** Oscillatory rheology and circular dichroism measurements and SEM images of PA nanofibers prepared with standard procedure, without annealing, to compare mechanical and structural differences between standard and annealing protocol. A) Time sweep measurement with oscillatory rheology, B) Circular dichroism spectra of PA groups, C) SEM images of LM/E-PA and D) LM/FN-PA.


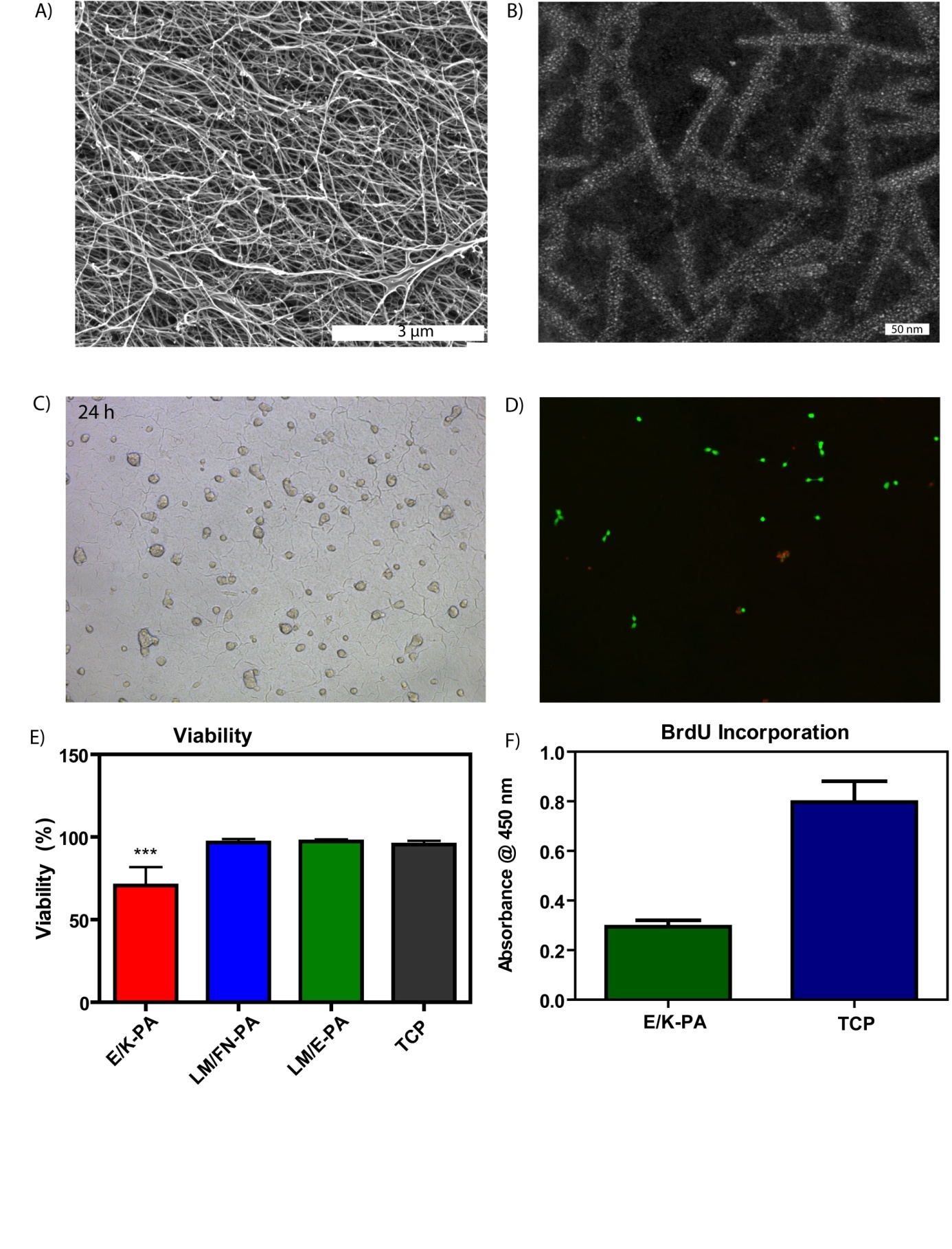


**Figure S3.** Morphological structure of E/K-PA gel and cellular viability and proliferation of C2C12 cells on control peptide group. A) SEM image of E/K-PA, B) STEM image of E/K-PA nanofibers; and low viability of cells on E/K-PA: C) optical microscope image of C2C12 cells on K-PA/E-PA coating, 10X objective, D) Live (green) and dead (red) cells stained with live/dead assay.


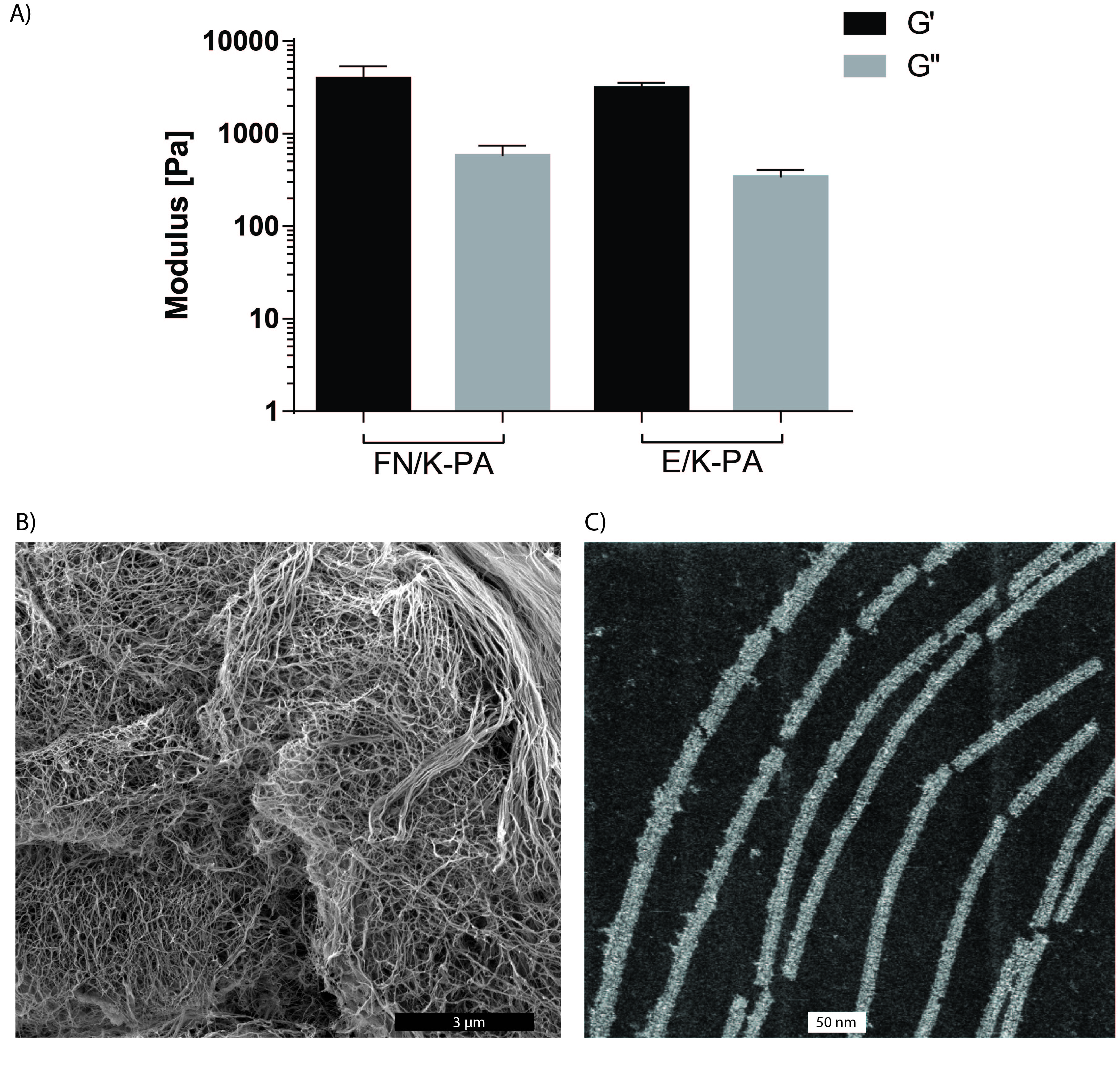


**Figure S4.** Characterization of FN/K-PA and E/K-PA groups. A) Time sweep measurement of FN/K-PA and E/K-PA by oscillatory rheology, B) SEM and C) STEM image of FN/K-PA group, prepared by using annealing procedure.


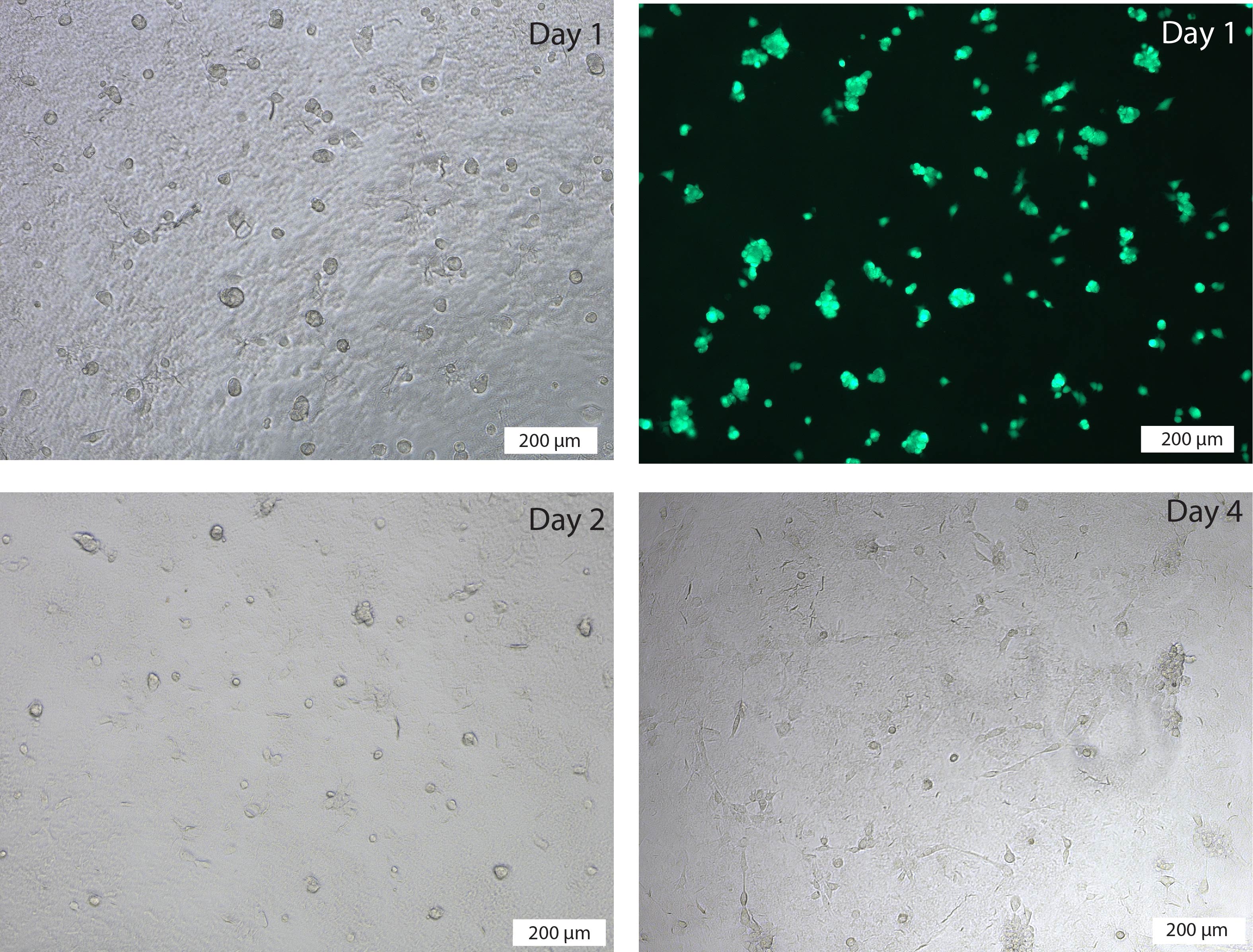


**Figure S5.** Morphology of C2C12 cells grown on FN/K-PA coated surfaces. Bright-field and fluorescence images clearly show that although initial adhesion and viability are high, cells forms clusters on top of FN/K-PA surfaces even at day 1.


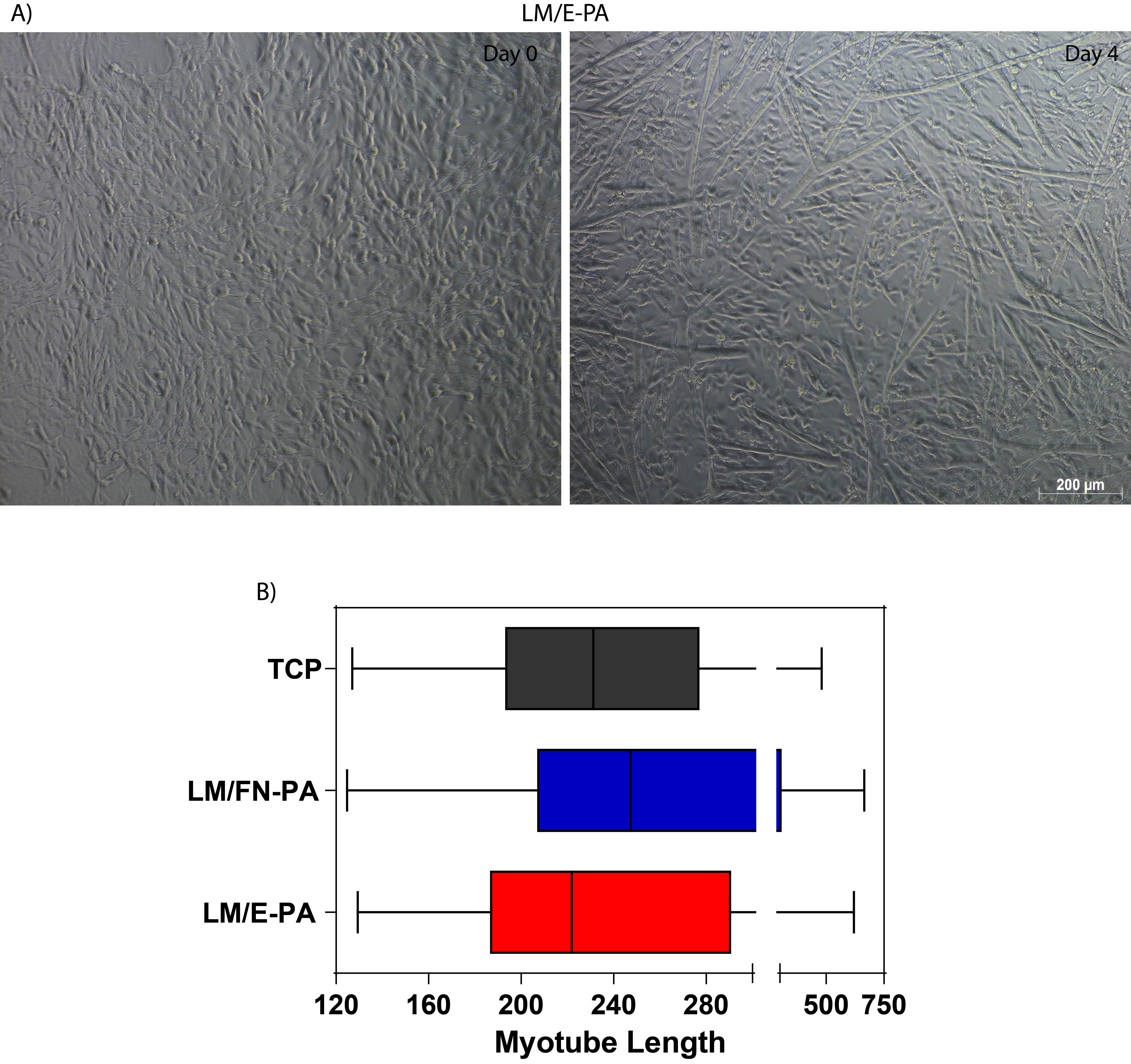


**Figure S6.** Myoblast and myotube morphology and length distribution of myotubes within different groups. A) Phase-contrast images of C2C12 cells before and after differentiation on LM/E-PA coated surface. Day 0 represents the differentiation induction day. B) Myotube length distribution. 180 myotube/group were measured and average myotube length was around 240 µm for all groups, and all groups had myotubes longer than 600 µm.
